# Challenges faced by parents in preventing online child sexual exploitation and abuse: a mixed methods systematic review

**DOI:** 10.3389/fpubh.2026.1765426

**Published:** 2026-02-17

**Authors:** Neelam Punjani, Amber Hussain, Wenting Yan, Lisa Hartling, Shannon D. Scott, Farah Elgaweesh, Megan Kennedy

**Affiliations:** 1 Faculty of Nursing, University of Alberta, Edmonton, AB, Canada; 2 Faculty of Medicine & Dentistry, University of Alberta, Edmonton, AB, Canada; 3 Sperber Health Sciences Library, University of Alberta, Edmonton, AB, Canada

**Keywords:** adolescent health, challenges, children, online child sexual abuse and exploitation (OCSEA), parents

## Abstract

**Introduction:**

Concerns about online child sexual exploitation and abuse (OCSEA) have been raised in society. Compared to adults, children and adolescents are more vulnerable as they are less able to assess risks and consequences. Prevention remains the most effective strategy for protecting children, and parents play an important role. However, many parents face barriers in recognizing and preventing OCSEA. This systematic review aims to identify parents' key challenges, ultimately informing more effective, evidence-based interventions and policy development.

**Methods:**

A comprehensive search was conducted in Medline, EMBASE, PsycINFO, CINAHL, Scopus, and the Cochrane Library. The search was conducted on March 20, 2025, covering articles published after 2018. Covidence was used for screening, with two independent reviewers and discrepancies resolved by a third. Data extraction was conducted independently by two reviewers and verified by a third. Methodological quality was assessed using JBI Critical Appraisal Tools. Findings were presented in tables alongside thematic analysis. Reporting followed PRISMA guidelines. The review was registered on OSF.

**Results:**

Nineteen studies were included, with qualitative (*n* = 9), quantitative (*n* = 6), and mixed methods (*n* = 4), covering children from different regions. A convergent integrated approach was used for synthesis. Challenges were categorized into six themes: 1) limited parental knowledge and awareness of OCSEA risks, 2) barriers to parent–child communication, 3) challenges in mediation strategies, 4) technological disparities and digital literacy gaps, 5) sociocultural influences on parental responses, and 6) inadequate support systems. These challenges were examined in terms of direct difficulties parents encounter and broader systemic influences. We emphasized how these challenges interact and reinforce one another.

**Conclusion:**

This review synthesized evidence on challenges parents face in preventing OCSEA, revealing a complex interplay of individual, cultural, and systemic barriers. By understanding these challenges, we advocate for a more comprehensive prevention system to support parents as the first line of defense.

**Trial registration:**

https://osf.io/fru5k/OSF, identifier: osf.io/fru5k.

## Introduction

Digital use among children and adolescents has been increasing over the years (1). In this digital age, children interact frequently with online environments through social media platforms, virtual communities, and digital learning tools. As a result, they are becoming increasingly reliant on digital technologies, which increases their exposure to various online risks (2). In this situation, parents are often left with the responsibility of monitoring these risks, but many of them feel underprepared (3).

Concerns about children's exposure to the risk of online harm have been raised across the world (4). Compared to adults, children and adolescents are more vulnerable to online risks and are more likely to take risks, as their brains are not yet fully developed, particularly the prefrontal cortex, which is responsible for assessing risks and considering consequences (4, 5). Therefore, they are more likely to be potential victims and have less capacity to judge what is right or wrong. A common concern in recent years is online child sexual abuse and exploitation (OCSEA) due to increased internet use, which includes online solicitation, grooming, viewing pornographic materials, forced or unwanted sexting, image-based abuse, sexual extortion, and exploitation (6–8). Unlike traditional forms of child sexual abuse and exploitation, OCSEA occurs online or through information and communication technologies (ICTs), bringing unique challenges that are particularly related to perpetrators, victims and social context in which OCSEA can take place (9). Perpetrators or offenders can use the internet and rapidly changing online technologies to anonymously befriend and groom children or adolescents they do not know in real life, with the intent to sexually abuse and exploit them both online and offline (10).

In the United States, the prevalence of the production, viewing, and distribution of child pornography has increased, with more than 78,000 unique web addresses containing child sexual abuse imagery (11). In Canada, between 2014 and 2022, nearly 72% of all reported child pornography cases involved the creation or distribution of such material, with more than 45,000 incidents of online child pornography (12). In Southeast Asia, the Disrupting Harm project revealed that in countries like the Philippines and Thailand, up to 20% of children had experienced online sexual exploitation and abuse, often initiated by strangers online (13). Meanwhile, Latin American nations like Brazil are reporting an increase in image-based abuse, but cultural stigma limits disclosure and prevention efforts (14). Sexting, defined as sending a text containing a nude or sexually explicit image, is also becoming more common as more young people use cell phones for texting (15). Meanwhile, for children, most of these situations are unwanted and unsolicited. For example, online solicitation, defined as the requests by a peer or adult to engage in unwanted sexual activities or sexual talk online, affects children as well (16). Online solicitation alone affects roughly 11% of young people (17). Children are often solicited to engage in risky online behavior or even sexual interaction (18). These trends all highlight an urgent need for actual actions to protect children from these risks.

Despite the serious risks, the prevalence of OCSEA has been largely unreported (19). Studies have found that children are hesitant to tell their parents if OCSEA occurs, due to threats from perpetrators and a natural fear of being blamed (6, 20). Furthermore, many are unsure of how or where to report these incidents, especially given that OCSEA is a comparatively new concept compared to traditional physical child sexual abuse (CSA) and child exploitation abuse (CEA), and the justice system in different countries lacks the capacity and resources to respond to OCSEA cases (19). Additionally, as technology continues to develop, more types of OCSEA are emerging, and there is no one-size-fits-all solution to these cases. Overall, this makes OCSEA harder for parents to identify and control.

OCSEA can also have a severe negative impact on the mental well-being of children, including stress responses, depressive symptoms, and post-traumatic stress disorder (PTSD) (6, 21, 22). ICTs provide perpetrators with new means to find, control, threaten, and intimidate victims, which can lead to the continuation and repetition of sexual harm (22). Another challenge of OCSEA is that it is difficult to distinguish whether the child's behavior is intentional or unintentional due to its persuasive nature. Especially in grooming situations, children are less likely to realize their victimization (23). In OCSEA cases, perpetrators may utilize legal gray areas and outdated legal frameworks to delay accountability. Moreover, existing laws' response tends to lag behind the emergence of new technologies used as tools of abuse. Thus, prosecution becomes increasingly complex and drawn out, especially in cross-jurisdictional contexts (24). By the time a final adjudication is reached, the psychological and developmental harm to the child may already be irreversible.

Given these challenges, prevention remains the most effective strategy for protecting children from OCSEA (19). Over the past two decades, several internet safety and education programs have appeared with stronger advocates for more preventive intervention (9). However, more re-evaluation and stronger evidence are needed to prove their effectiveness and support better incorporation into practical strategies (25, 26). Among these initiatives, parental roles are emphasized: parents are seen as the first safeguard for their children, expected to control and supervise online activities while developing a better understanding of risks (16, 21, 27, 28). Garazi and colleagues have claimed that parental monitoring and supervision can serve as protective factors for children (29). In other words, if parents can detect OCSEA promptly, they can reduce its occurrence. However, there remain several barriers and challenges that parents face in preventing OCSEA.

Many parents struggle to effectively supervise their children's internet use or communicate with them, particularly in the context of rapidly developing ICTs. Therefore, traditional approaches for preventing child sexual abuse and child sexual exploitation may not be sufficient to detect or address these risks. While some studies have highlighted the importance of the parental role and discussed certain difficulties in preventing OCSEA with rising concern, a comprehensive understanding of the weak points and challenges parents face, and what kinds of parental prevention may be effective, is still lacking (21, 30, 31). Therefore, this work aims to conduct a systematic review to synthesize the existing evidence on challenges parents face with respect to OCSEA. Guided by the research question: what are the challenges faced by parents in preventing various forms of OCSEA, we hope the findings can inform more effective, evidence-based interventions and policy frameworks that strengthen parental capacity in preventing OCSEA in the future.

## Methods

This systematic review was conducted in accordance with the JBI methodology for mixed-method systematic review and adhered to the Preferred Reporting Items for Systematic Reviews and Meta-Analysis (PRISMA) framework (32). This review was conducted in accordance with an *a priori* protocol registered in OSF.

### Inclusion/exclusion criteria

#### Participants

This review considered studies that included participants who are parents, caregivers and guardians of children or adolescents who are under 18 years of age. Studies that only included children and adolescents and did not involve perspectives or experiences from parents were excluded. Studies that included only teachers or other professionals and did not capture parents' perspectives were also excluded.

#### Concept

This review considered studies that explored the challenges faced by parents, caregivers, and guardians in preventing OCSEA. According to Public Safety Canada, OCSEA refers to situations where children are tricked into viewing or participating in online sexual activities. This includes grooming, sexting, sextortion, capping, and the distribution of sexual images and videos (8).

For the purpose of this review, challenges in preventing OCSEA are defined as any potential barriers encountered in the everyday interactions between parents and their children, such as those related to communication, education, and parental involvement. Studies that did not address challenges or barriers related to OCSEA prevention, or that focused exclusively on law enforcement or education without involving parental perspectives, were excluded.

#### Context

The context of this review considered online or digital environments across various platforms including social media or online messaging apps in any cultural context.

#### Types of Sources

This systematic review considered published primary research studies employing quantitative, qualitative, and mixed methods designs for inclusion. Gray literature, such as student dissertations that are primary studies, was also considered for inclusion. The reference lists of the were reviewed to identify any additional primary studies that could be considered for inclusion. Review papers, opinion pieces, blog posts, and editorials were also excluded.

#### Search strategies

Text words from article titles and abstracts, as well as index terms and Medical Subject Headings [MeSH], were used to develop a full search strategy. This was adapted for each database and information source by an experienced librarian (MK). The search was undertaken on March 20, 2025 (Refer to Supplementary File A for full search strategies). In addition, the reference lists of the selected articles were screened for additional relevant papers.

Only sources published in English were included due to resource restrictions. Sources published from 2018 were included to capture the most current evidence following key global developments in online child protection, including the adoption of the WeProtect Global Alliance's Model National Response and UNICEF's call for coordinated action in child online protection (33, 34). This timeframe also includes the COVID-19 pandemic period, both pre-pandemic and pandemic-related shifts in related challenges.

The databases that were searched included Medline, EMBASE, PsycINFO, Cumulative Index for Nursing and Allied Health Literature (CINAHL) via EBSCOhost, Scopus via Elsevier, and the Cochrane Library via Wiley.

#### Source of evidence selection

All identified records from the search were uploaded into Covidence (35), a web-based tool for systematic review management. Duplicates were automatically removed by Covidence. Then title and abstract screening was conducted independently by two reviewers (WY and FE). Potentially relevant studies were moved to full-text screening for decision of final inclusion. Full-text studies were reviewed by the same reviewers (WY and FE). Full-text studies that did not meet the inclusion criteria were excluded. Any disagreements in the screening process were resolved by discussion with a third reviewer (AH or NP) with expertise in both review methodology and adolescent health.

#### Data extraction and critical appraisal

Data were extracted from the selected papers by two independent reviewers (WY and FE) with the third reviewer (AH) verifying all the extracted data. A data extraction sheet was developed by the research team and piloted using 10% of the included studies. Following the piloting process, the researchers who were involved in data extraction held meetings to add/edit items to the sheet to improve its comprehensiveness. Ultimately, the data extracted from each study included bibliographic details (authors, year of publication, and country of research), the study's aim, design (quantitative, qualitative, or mixed method), population characteristics (sample size and parent type: mother, father, or both), as well as the key findings (refer to Supplementary File B for extraction table). Types of OCSEA discussed and the study's research questions were recorded as well. Any discrepancies were identified and resolved through discussion with other reviewers in the team (NP and AH).

The methodological quality and potential risk of bias of included studies were evaluated using the Joanna Briggs Institute (JBI) Critical Appraisal Tools (36). These tools consist of 8–11 criteria that are rated as “yes,” “no,” “unclear,” or “not applicable.” Because the publicly available JBI checklists do not cover mixed methods studies, we combined criteria from both the JBI qualitative and quantitative checklists. In this review, we categorized study quality as high (≥75% criteria met), moderate (50–74%), or low (< 50%) (70). Quality appraisal was carried out by two reviewers (WY and AH), with both reviewers assessing all included studies, discrepancies were solved by a third reviewer (NP). Critical appraisal results did not lead to exclusion of studies, but they influenced the synthesis by weighting interpretations with greater caution for findings from studies rated as moderate or low quality using the JBI tools.

#### Data analysis and presentation

A narrative description of the included studies is provided to offer an overview of all the relevant studies. All extracted data are presented in tables that follow the format of the data extraction sheet, ensuring consistent reporting of the information obtained from the included studies. Data from the selected studies were then synthesized with a convergent integrated approach, which means qualitative and quantitative data will be combined for data transformation. By using this approach, findings from quantitative studies were converted into a qualitative form for synthesis, which means data in quantitative studies were transformed into textual data (37). This stage is also known as “qualitizing”—a stage of providing a narrative interpretation of the quantitative results.

After assembling all the qualitative data and textual description of results from quantitative studies, we then employed thematic analysis guided by the review questions to present the findings of this research (38). After reading through all the included studies and having all the extracted data, initial codes were created by categorizing the similar findings together by the team. Then the research team collaboratively developed an initial thematic framework with some broad themes by merging the initial codes. As coding went on by the research team, new themes or secondary themes were added to the framework, and earlier studies were checked again using the updated version. In the end, the developed themes were further refined and given names through discussion. Any discrepancies that happened in the development of the thematic framework were resolved by team researchers, ultimately reaching an agreement.

## Results

### Study -description

**Figure 1:**
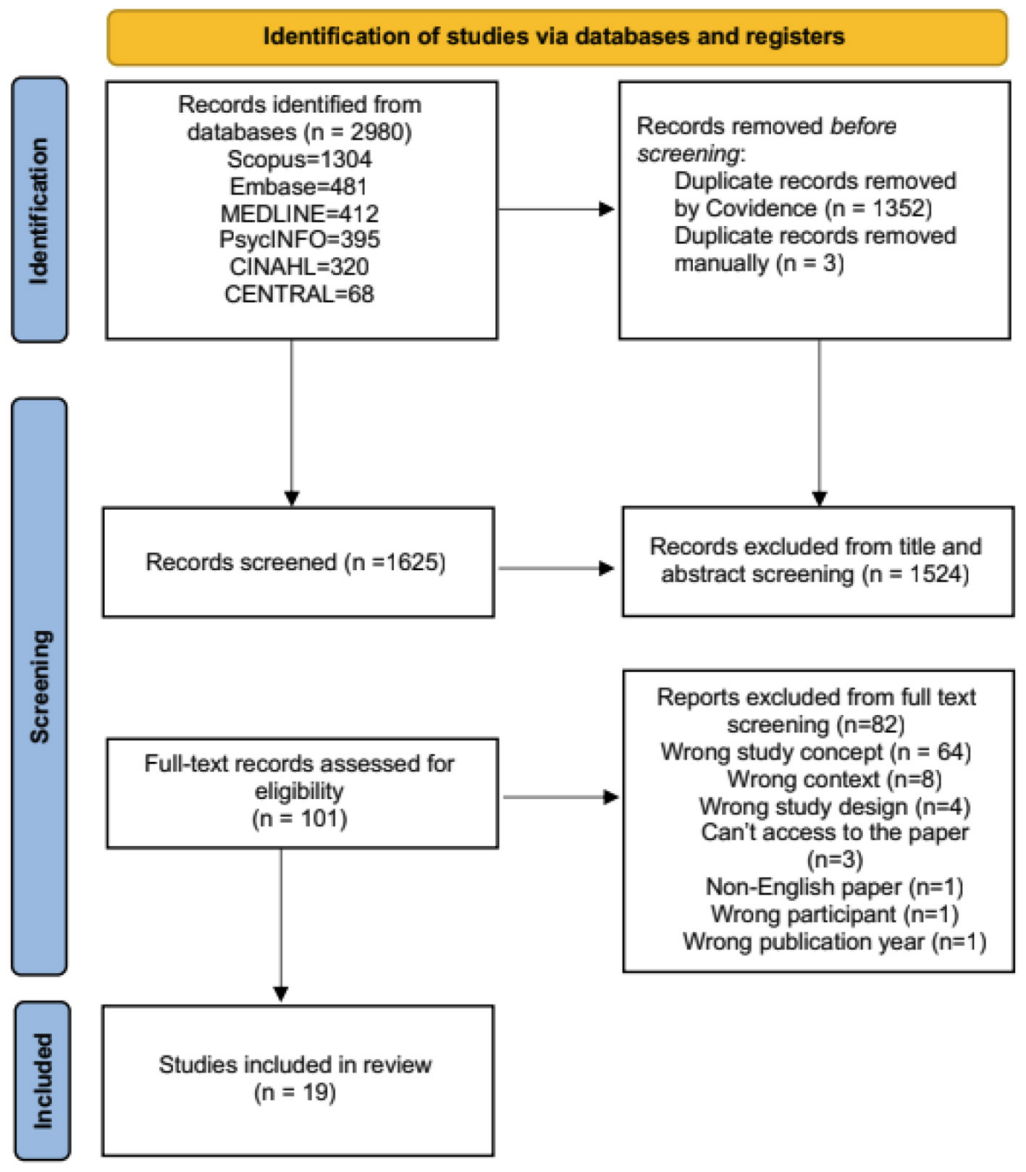
Study selection process.

Nineteen studies were included from 2018 to 2025. The study selection process is detailed in Figure 1. Out of 19 studies, 17 were classified as high quality, while two were classified as moderate quality. None of them were rated as low quality. The detailed results of these assessments are provided in (Supplementary File A).

The studies were sourced from different countries: four from the USA (39–42), five from Southeast Asia (43–47), two from Western Asia (48, 49), four from Western Europe (50–53), one from New Zealand (54), one from East Asia (55), one from West Africa (56), and one conducted in Australia and the United Kingdom (57) recruiting participants from more than one country.

Most studies involved both fathers and mothers as primary participants, with two studies focusing exclusively on mothers (45, 47). In total, there were nine qualitative (41, 43–47, 49, 53, 54), six quantitative (42, 52, 55–58), and four mixed methods studies (39, 40, 50, 51).

The types of OCSEA included sexting (41, 42, 48, 53), cybergrooming (46), online child sexual abuse (45, 47, 57), online pornography (43, 44, 49, 50, 52, 54, 55), with several studies examining multiple aspects of OCSEA (39, 40, 51, 56). While definitions and emphases varied slightly across studies, all fell within the review's inclusive conceptual framework, and parental challenges were synthesized thematically across behaviors.

### Findings

The systematic review highlighted that limited parental knowledge, communication barriers, and sociocultural influences hinder effective parental engagement in preventing (OCSEA). The six key themes and their subthemes summarize the challenges parents face, as shown in the Table 1.

**Table 1:** Identified themes.

**Theme → **	**Subthemes**	**Key citations**
1. Limited parental knowledge and awareness of OCSEA risks	1.1 Lack of awareness about specific OCSEA behaviors 1.2 Misconceptions about OCSEA perpetrators and victims	(41, 43, 45, 46, 50, 55, 57)
2. Barriers to parent-child communication on online sexual risks	2.1 Discomfort and embarrassment in discussing sexuality 2.2 Perceived child resistance to parental guidance	(39, 41, 42, 46, 49, 50, 52, 57)
3. Challenges in parental mediation strategies for online safety	3.1 Authoritative vs. permissive parenting style 3.2 Usefulness and limitations of mediation 3.3 Gender differences in mediation Practices	(41, 42, 45, 47–50, 52, 55–57)
4. Technological disparities and digital literacy gaps	4.1 Generation Gap—Parents as “Digital Immigrants” vs. Children as “Digital Natives” 4.2 Rapid evolution of technology	(39, 41–43, 46, 51, 53)
5. Sociocultural and contextual influences on parental responses to OCSEA	5.1 Cultural taboos and social norms 5.2 Gendered perceptions and double standards 5.3 Family dynamics and resource constraints	(41, 45, 48–50, 52, 53, 56)
6. Inadequate support systems and resources for parents	6.1 Lack of formal resources and training 6.2 Reliance on informal or unreliable sources 6.3 Need for professional involvement	(39, 40, 46, 47, 52, 54)

### Theme 1—Limited parental knowledge and awareness of OCSEA risks

This theme captures the significant gap in parents' understanding of OCSEA, including the nature of online risks, the prevalence of exposure, and the specific behaviors associated with it. Across different contexts, from Pakistan to the United States, parental awareness emerged as a foundational determinant of whether communication about OCSEA occurs, hindering proactive engagement and effective protection of children.

#### Subtheme 1.1—Lack of awareness about specific OCSEA behaviors

A large proportion of parents across various studies lacked comprehensive awareness of online risks, including exposure to pornography, sextortion, and exploitative relationships (45, 46, 55). This lack of knowledge was found to be a challenge in both developing and developed nations. For example, a study in Pakistan found that parents had a moderate level of awareness but struggled to identify accidental exposure (43), while a study in Malaysia found that 40% of parents were unaware of the term “online grooming” (46).

Even when awareness was present, limited digital literacy constrained parents' ability to take appropriate action to protect children. Parents in Ireland, the USA, and Malaysia reported struggling to keep up with technology, which impacted their ability to make informed parenting decisions regarding online control and to initiate meaningful conversations (41, 46, 50). Some parents, like those in a study from Delhi, India, felt particularly helpless due to low education and limited access to technology, believing risks were inevitable or that their children would ignore their guidance (45). This awareness-action gap directly influenced the frequency and quality of parent-child communication, as many parents reported feeling paralyzed due to uncertainty or low confidence (45, 50). Although they realize that smartphones and the Internet are important in their children's lives, many parents don't know how to prevent online grooming (45, 46). Some expressed that they have no choice but to give their children such devices (45), while others only monitored or tried to limit their children's internet use to protect their children from online predators (46).

#### Subtheme 1.2—Misconceptions about OCSEA perpetrators and victims

Misconceptions about who perpetrates and who is at risk from OCSEA were a widespread issue (45, 57). Pandey & Reddy (45) noted that mothers often underestimated non-penetrative online sexual abuses, focusing only on penetrative acts, such as rape or sodomy, as sexual abuse since the perpetrators did not have physical access to their children. Thus, they believed it did not require serious police intervention.

Similarly, these mothers believed perpetrators were primarily uneducated, poor, young males who were “strangers” to the child (45). In a more general context, a study in Australia and the UK found that parents were worried that telling children that family members might be perpetrators could lead to negative consequences (57). The tendency to overlook the severity of non-physical harm and to cling to stereotypes about perpetrators limits protective efforts across different contexts.

### Theme 2—Barriers to parent-child communication on online sexual risks

Communication was identified as a pervasive challenge across all studies, regardless of country or culture. It highlights the challenges parents face in initiating and maintaining open, effective communication with their children about online risks and sexual content. Parenting style, digital competence, cultural norms, and a lack of institutional support collectively influenced the likelihood and quality of communication.

#### Subtheme 2.1—Discomfort and embarrassment in discussing sexuality

Parental discomfort with sexual topics, often rooted in their own upbringing or social taboos, emerged as a major inhibitor to dialogue in every study. This discomfort was an important factor for parents in the US, UK, Ireland, and Malaysia (41, 46, 50). Culturally specific taboos within certain communities hindered sexual discourse, as it was perceived as forbidden and inappropriate (49). This created cultural and psychological barriers for adolescents to openly discuss sexuality and related issues with their parents or vice versa (49). Even parents who were aware of online grooming were generally uncomfortable with discussing sex with their children (39, 41, 46).

This discomfort was often more pronounced for specific topics (57). Parents in Ireland reported difficulty defining pornography in an age-appropriate way, leading them to avoid the topic entirely (50). One parent stated, “Some of the content would be too explicit for adults, let alone children, now how do I explain the concept behind this...” (50), while another stated, “not the right time, still too young, sensitive topic” (46). Likewise, many adolescents do not discuss issues related to sexuality with their parents or teachers because they feel embarrassed or perceive them as insufficiently knowledgeable (52).

#### Subtheme 2.2—Perceived child resistance to parental guidance

Parents have a belief that older children and teenagers would not be receptive to guidance on sensitive topics due to conflicting external peer pressures. Across studies in the USA and Ireland, parents expressed concerns that peer norms and online behaviors often countered parental guidance, and adolescents were more likely to accept behaviors normalized by their online communities than those discussed at home, making it harder for parents to instill their perspectives on appropriate online conduct (39, 42, 50). This sentiment, that adolescents perceived their parents as unable to understand the sexual lives of young people, was a common barrier to effective communication (50). Even when parents initiated the conversations, some were immediately faced with resistance from their children due to discomfort (50).

### Theme 3—Challenges in parental mediation strategies for online safety

This theme focuses on the challenges parents face in implementing effective mediation strategies to prevent OCSEA, highlighting how the effectiveness of these strategies varies across different contexts and family dynamics. There are two main parenting styles discussed in the studies: authoritarian involved strict control exerted on children without open discussion; on the other hand, permissive parents may avoid initiating difficult conversations. There were additional parental mediation strategies discussed, including discursive, investigative, and gatekeeping tactics to talk about sexting. Discursive strategies include using examples to prompt discussion, being open and direct, and gradually introducing the topic. Investigative strategies include monitoring children's digital content and self-educating about the media their children use. Gatekeeping strategies include enforcing specific rules to manage how and when children engaged with media.

#### Subtheme 3.1—Authoritative vs. permissive parenting style

Findings from studies in Israel and the US consistently showed that parenting style is a contributing factor. An authoritative parenting style, characterized by a balance of control, open communication, and accurately assessing the severity of children's sexting activities and engaging in such behaviors, was linked to positive outcomes in parent-child communication about sexting (48).

In contrast, both authoritarian (high control, low discussion) and permissive (low control, low discussion) styles were positively associated with dysfunctional communication and increased risk of exposure to online dangers (42, 48).

#### Subtheme 3.2—Usefulness and limitations of mediation

The usefulness of specific mediation tactics showed mixed results across studies. A study in Israel found that technologically restrictive mediation (e.g., parental controls) increased sexting behaviors, suggesting a backlash effect (48). However, a different study in the US found no significant association between technological control and sexting (42).

The most consistent finding was the benefit of a combined approach. Corcoran et al. (42) suggested that a combination of restrictive mediation and active mediation (discussing and jointly engaging in internet use) was associated with a lower likelihood of youth sending and receiving sexts. On the other hand, permissive parenting led to lower parental mediation of sexting despite perceived susceptibility to sexting, and this increased children's exposure to online risks (48, 57). This suggests that while restricting access alone may not be effective, it can be a useful tool when paired with open, discursive communication.

#### Subtheme 3.3—Gender differences in mediation practices

Gender of parent and child played an important role in parental mediation practices. Several studies, including those from the UK, Pakistan, and Israel, found that mothers predominantly favored restrictive and active mediation, setting rules and discussing risks, particularly for girls compared to boys (49, 56) with the belief that most victims are females (45). This can lead them to underestimate the importance of mediating their sons' online activities (45). However, this gender disparity was not noted in all studies. Studies showed that mothers are aware of the risks of the internet, regardless of gender, and caution is required when raising boys and girls, and that they are the primary educators for both sons and daughters (47, 52).

Speno and Halliwell (41) found that parents' and children's gender hugely influenced the comfort of parents to initiate conversations. Studies noted a double standard, with mothers being more lenient toward boys‘ porn viewing but having a “sweeping prohibition” for girls (49). In contrast, fathers more often relied on technical controls such as device settings or monitoring software for both their boys and girls (56). A study in China found that while fathers were more likely to be unaware of a child's violence/pornography exposure, mothers were more likely to engage in sex-related communication with a child (55). This is reinforced by a study in Ireland, where fathers believed it was the mother's role to talk about sexual health with their daughters (50). While these gender disparities were not found in all studies, the pattern suggests a common division of labor in parental mediation.

### Theme 4—Technological disparities and digital literacy gaps

This theme addresses the technological challenges parents face in protecting their children from OCSEA, including their limited digital literacy and the rapid evolution of technology. Parents often lack the technological proficiency to keep up with their children's online activities, creating a significant barrier to effective monitoring and protection. Furthermore, the accessibility and privacy of technology make it challenging for parents to monitor their children's online interactions effectively, especially due to its fast-paced nature.

#### Subtheme 4.1—Generation gap—Parents as “Digital Immigrants” vs. children as “Digital Natives”

The generational gap was evident by many parents across different countries reporting lower digital literacy than their children, leaving them ill-equipped to supervise online activity or recognize warning signs (41, 46). This lack of knowledge was a primary reason parents felt “at least one step behind” their children and felt their guidance would be ineffective (41). As noted in a study from the UK, this generational divide requires parents to move beyond traditional sex education to effectively address online risks (51). Lamprianidou et al. (53) used the term “digital immigrants” to describe parents' general limited understanding of technology or misuse of social media in sexting. This can hinder effective conversations with their children about issues related to sexting, smartphones, and social media (41).

Furthermore, adults reported feeling behind children in applying online parental safeguards, as children can easily circumvent them (51). There is a higher level of complexity that digital media introduced to sexual education, requiring parents to go beyond traditional talk to educate their adolescent children about risks of sexual activity online (41).

#### Subtheme 4.2—Rapid evolution of technology

A compounding factor is the rapid evolution of technology, which makes it difficult for parents to stay knowledgeable about risks and mediation techniques (42). This rapid change has increased the likelihood of accidental exposure to inappropriate content, particularly for younger children, and has decreased opportunities for proactive dialogue (39, 43). This challenge requires parents to adapt quickly, as evidenced by a study in Indonesia, which noted the need for parents to be knowledgeable about constantly changing online dangers (47).

Conversely, the “generation gap” suggests a barrier in communication because youth may find it difficult to talk to adults about online issues, believing “They will ask too many questions. It's so annoying.” (41, 50)

### Theme 5—Sociocultural and contextual influences on parental responses to OCSEA

This theme demonstrates how parental responses to OCSEA are shaped by cultural and social factors, showing both universal challenges and context-specific norms. Cultural and social norms around sexuality and technology use can inhibit parents' ability to engage in OCSEA prevention effectively. Additionally, gender-based stereotypes and double standards influence how parents perceive and address OCSEA risks for boys vs. girls.

#### Subtheme 5.1—Cultural taboos and social norms

Social and cultural norms were a major influence on parental engagement across the world. One finding was the “culture of silence” surrounding OCSEA, where parents would underestimate online and non-penetrative sexual abuses (45). This was most acutely seen in studies from Israel, where cultural taboos created significant barriers to open sexual discourse, with mothers fearing negative reactions from fathers if they discussed sexuality (49). Another study from Israel further reinforced this, noting that cultural conservatism led some parents to avoid discussions entirely, especially between fathers and their daughters (48). This was not only limited to Eastern countries, Dawson et al. (50) found that fathers of adolescent girls believed that, even though they would be interested in having discussions with their daughters, it was the mother's role to talk with their daughters about issues related to sexual health.

#### Subtheme 5.2—Gendered perceptions and double standards

Parental mediation of online risks is heavily influenced by gendered stereotypes and double standards, creating a consistent pattern across the studies. Mothers are often seen as the primary mediators, but they show a bias in their protective efforts, frequently exhibiting a “sweeping prohibition” and greater monitoring for girls while “turning a blind eye” to similar behaviors, like pornography viewing, by boys (49).

This is often rooted in the perception that girls are more vulnerable to social stigma from “sexting gone wrong,” while boys are seen as more likely to be the perpetrators or consumers of pornography (52, 53). This disconnect between awareness and action means that even when parents recognize the differing consequences for each gender, they may still adopt a less effective “gender-neutral” approach. In contrast, fathers who engage in mediation tend to rely more on technical controls and monitoring software rather than open communication, further highlighting the gendered division of labor in addressing online safety (56).

#### Subtheme 5.3—Family dynamics and resource constraints

Family dynamics, such as time constraints and work-life balance, were also reported as a factor impacting a parent's capacity to address OCSEA (46). The challenge of single parenting and the demands of full-time work additionally made it difficult for parents to find the time and energy for these discussions (41).

### Theme 6—Inadequate support systems and resources for parents

This theme underscores the lack of accessible, formal resources and professional support to help parents navigate OCSEA prevention, leaving them reliant on informal or unreliable sources. Parents often lack access to formal training or resources on OCSEA prevention, leaving them ill-equipped to address risks effectively. In the absence of formal resources, parents often turn to informal sources like social media or peers, which may provide inconsistent or unreliable guidance.

#### Subtheme 6.1—Lack of formal resources and training

Parents indicated a desire for greater involvement from schools, either to complement or take on the responsibility of sex education altogether. Across studies in the US, UK, Spain, and Italy, parents expressed a strong desire for greater involvement and guidance from formal institutions like schools and healthcare professionals (39, 46, 52). Yet, studies reported minimal integration between schools and parents, leading to fragmented or contradictory messages (46, 52). The lack of training for both parents and teachers in online security and affective-sexual education, internet use, and pornography consumption is a gap (52).

#### Subtheme 6.2—Reliance on informal or unreliable sources

In the absence of formal support, parents often turned to informal resources such as social media and other parents for guidance, which varied widely in quality and reliability (39). This reliance on inconsistent information meant that parents' understanding of online risks often depended on their own limited experiences, leading to inconsistent education for their children (54).

#### Subtheme 6.3—Need for professional involvement

Parents recommended the need for professional guidance, such as training from school staff, nurses, or social workers, to help them understand mobile technologies in addressing online sexual risks and practical tools for engaging with their child (39, 46). This highlights a critical need to equip families and teachers with the standardized tools and professional knowledge necessary to discuss issues related to sexuality and online safety at home and at school.

Particularly in porn literacy, Healy-Cullen et al. (54) found that parents believe that education requires “professionals” to deliver it, as parents may feel biased or uncomfortable. Furthermore, Saleha et al. (47) suggested that maternity nurses should promote reproductive health education, indicating a need for professional support in parenting strategies.

## Discussion

This review has identified various challenges that parents encounter in their efforts to prevent OCSEA. The findings revealed that parents made efforts to protect their children from being exposed to OCSEA. However, many lacked a basic understanding and awareness of OCSEA. Improving awareness of online sexual abuse is the most immediate way to support prevention (59). In our findings, some parents were unfamiliar with the full scope of the concept and did not know what types of sexual abuse or exploitation fall under the category of OCSEA, as many were unaware of issues such as online pornography and grooming. This lack of awareness hinders their ability to recognize when their children are in danger and prevents them from taking timely action to stop the occurrence of OCSEA.

Additionally, the online nature of OCSEA often leads to parents' underestimation of the threat. Physical or offline sexual abuse and exploitation are easier to detect and are strictly regulated by law, whereas OCSEA is less visible due to the various devices and applications in the online digital environment (9). Parents tend to believe that such abuse is unlikely to affect their children, especially since they live with them and assume there is no opportunity for their children to get exposed. This is also referred to as the “not my child” phenomenon in Davis et al.'s (60) study, where parents believed that by relying on their honest relationship with their children, their child would not engage in risky behaviors that could lead to OCSEA. In reality, this belief and limited concern can create opportunities for OCSEA offenders. Meanwhile, research indicates that OCSEA perpetrators are more likely to be younger and have higher levels of education and socioeconomic status, however many mothers still believe that offenders are typically low-educated strangers, thereby letting their safeguard down (11, 16). Low awareness of the risks and existing misconceptions further creates opportunities for offenders and highlights the challenges of identifying the occurrence of OCSEA faced by parents.

Even when parents are able to understand and identify OCSEA, the next challenge is how they can foster open and effective communication with their children. In our review, participants in several studies reported feelings of “embarrassment” and “discomfort” when discussing sex-related topics with their children. This discomfort creates barriers to discussing sexual abuse with children and having effective conversations (61). Parents struggle to find appropriate language to initiate these conversations and are concerned that using explicit terms may frighten their children, especially considering their young age and emotional immaturity. These findings align with previous research suggesting that dysfunctional sexual conversations between parents and children may paradoxically increase the exposure to online risks, possibly due to misinterpretation or lack of constructive dialogue (48).

Moreover, the communication barrier is not one-sided. Parents also reported resistance from their children who also experience embarrassment and discomfort during such discussions. Children often feel more relaxed and receptive when discussing topics related to sexuality with their peers rather than with their parents. Children and adolescents are more likely to disclose experiences of sexual abuse to peers before approaching their parents (62). Additionally, even when parents are willing to have appropriate and informative discussions about the risks of online sexual abuse and exploitation, these conversations often end quickly and lack depth, which reduces their effectiveness.

The initiation and usefulness of communication are also impacted by parenting styles and mediation strategies. In our review, we discussed the influence of two parenting styles (authoritative and permissive) and three primary mediation types (restrictive, active, and technical). Different parenting styles and mediation strategies bring unique challenges to communicating effectively and preventing the occurrence of OCSEA. For example, parents with a permissive parenting style may try to avoid embarrassing and uncomfortable topics like sexting, resulting in weaker mediation and low-quality communication (57, 63).

Meanwhile, fathers, who are more likely to rely on restrictive or technical controls to limit children's device access, were sometimes reported as counterproductive, as they tend to engage in less active mediation strategies than mothers (63). Mothers often used restrictive and active strategies more with daughters, underestimating risks that their sons may face. Some other mediation strategies were found to be potentially useful, but they require parents to be confident and equipped with certain knowledge, which connects back to the earlier challenges we discussed, such as lacking knowledge and feeling uncomfortable communicating. Overall, to overcome communication challenges and achieve a more balanced communication approach, our findings suggest that authoritative parenting combined with active and gender sensitive mediation may be most conducive to having effective communication with children.

A unique and repeatedly mentioned challenge of preventing OCSEA is the rapid development of technology (13). Media and digital literacy can help parents recognize potential risks and act for prevention. Conversely, technological complexity and the generation gap often hinder parents' ability to enhance their digital literacy. This leads to barriers to having effective supervision, mediation and communication. It highlights that while awareness of risks is important, as discussed, keeping up with emerging platforms and threats is a constant challenge for parents.

In our review, we also identified the important impact of social and cultural taboos. Our findings echoed studies from various countries revealing the diverse ways in which child sexual abuse is contextualized, as well as the variability in parental responses (64–67). The contextualization of child sexual abuse was constructed by Western society, which means that the concept and related interventions may not be applicable within the values or realities of other cultures or communities (66). Therefore, in some more conservative communities, cultural norms contribute to the silence around online sexual risks and reinforce gender disparities in communication and intervention. In some cases, information delivered in OCSEA prevention may not follow some of the cultural ethics (68). For example, while parents are encouraged to have open dialogues with their children about preventing potential OCSEA, cultural taboos in Arab society may cause mothers to fear negative reactions from fathers when engaging in such conversations (49). As a result, both cultural silence and gender bias create significant barriers to launching OCSEA prevention, even when it has been proven effective.

We also pointed out challenges at broader structural challenges that hinder parents' ability to protect their children from OCSEA. A recurring issue across studies was the absence of comprehensive, coordinated systems and policies designed to support parents. Across studies, parents expressed a need for stronger institutional infrastructure across schools and communities. They highlighted the need for integration between parents and schools but ultimately pointed out the reality that no related training or programs have been received. This contrasts with the large number of school-based prevention programs on CSA that have been implemented (69). While schools have been the primary setting for delivering CSA prevention programs, our review suggests they have not been utilized as an effective platform for providing structural support in OCSEA prevention.

Moreover, school-based education programs are not adequate. As advocated by other researchers, what we need is a more comprehensive and multi-level system of support (29). Schools often operate independently of families, with minimal collaboration, resulting in fragmented education and unclear responsibilities. The consequence of this is that parents tend to rely more on informal networks or unregulated online advice as we found in our review. Without accessible professional support or public resources, parents are left to navigate these challenges alone and often feel unqualified and unsupported.

### Future implications

The family is often viewed as the foundation of a bottom-up approach to preventing child sexual abuse (68). The challenges identified in this study could help inform future primary prevention strategies for OCSEA, particularly those targeting parents. Among the three levels of prevention (primary, secondary, and tertiary), primary prevention plays a particularly significant role and may help to break the vicious circle. We advocate a multi level primary prevention strategy: At the micro level, we can improve parental awareness and understanding of OCSEA, promote open and healthy parent-child communication, guide more effective parental styles and mediation strategies, and build confidence in navigating online safety in the digital world. Parental responses may need to be tailored to the specific type of OCSEA risk. For instance, prevention of sexting behaviors benefits from discursive strategies and peer-inclusive education, while cases involving online grooming or sextortion may require stronger digital literacy and use of monitoring tools. To support the practical application of these strategies across different levels and global contexts, we summarize key recommendations aligned with each of the six themes in Table 2. This table highlights implications for parents, children, and first responders (e.g., educators, healthcare professionals, government agencies) to ensure coordinated and actionable responses.

**Table 2:** Summary of recommendations based on six themes across stakeholder levels.

**Themes**	**Recommendations for parents**	**Recommendations for children & youth (in relation to parents)**	**Recommendations for first responders/ organizations/ government**
1. Limited parental knowledge and awareness of OCSEA risks	Build foundational knowledge of OCSEA forms (e.g., grooming, sextortion, image-based abuse); understand platforms and digital risks; recognize early warning signs.	Support age-appropriate awareness of online risks; feel safe seeking help without fear of punishment.	Provide clear, consistent public definitions of OCSEA; offer accessible resources for families; integrate OCSEA awareness into school and health curricula.
2. Barriers to parent-child communication on online sexual risks	Foster open, ongoing, and non-judgmental conversations about online safety and sexuality.	Feel safe to disclose online experiences; be empowered to express discomfort and ask questions.	Train professionals to promote open family dialogue; shift away from surveillance-only models.
3. Challenges in parental mediation strategies for online safety	Apply balanced mediation strategies (e.g., discursive, restrictive, and investigative); adapt style to child's needs; avoid over-reliance on authoritarian or permissive approaches.	Participate in open, trust-based discussions; understand rules and reasoning behind them.	Promote evidence-informed mediation guidance; provide parenting tools that combine control and communication.
4. Technological disparities and digital literacy gaps	Strengthen digital literacy on privacy, platforms, and safety settings; keep pace with evolving technologies.	Understand digital boundaries, permanence, and consent; build skills to navigate online spaces safely.	Launch digital literacy initiatives for families; partner with tech platforms for safer design and user education.
5. Sociocultural and contextual influences on parental responses to OCSEA	Reflect on how culture, stigma, and gender norms shape safety conversations; adapt parenting to context.	Balance cultural identity with personal safety; feel their background and values are respected.	Create culturally responsive and equity-driven programs; collaborate with local leaders and community networks.
6. Inadequate support systems and resources for parents	Identify and access formal support systems (e.g., schools, health services, legal resources); advocate for child protection within institutions.	Be supported in seeking help; know how to access relevant services with parental guidance.	Ensure clear, coordinated access to services; strengthen intersectoral collaboration across child protection, education, health, and justice sectors.

At the meso level, we can enhance stronger integration between families, schools, and community organizations and seek advice from healthcare and social service professionals, particularly in contexts where cultural or social taboos limit family dialogue; At the macro level, coordinated national and international policies are needed to ensure parents are not left to manage these challenges in isolation and that all stakeholders are on the same page regarding cross-sector collaboration. Furthermore, one-size-fits-all approaches should be avoided, and programs should be culturally sensitive, gender inclusive, and adaptable to different socioeconomic contexts.

We also identified that some of the OCSEA prevention challenges acknowledged in our study overlap with challenges previously identified in the context of CSA or CEA prevention. This overlap reveals that certain challenges, such as limited parental awareness, communication barriers, and the impact of cultural norms, are not unique to the online environment but are part of broader issues in preventing child abuse and exploitation. Therefore, the aim should be to ultimately empower parents as the first line of defense. And the next step might be integrating culturally sensitive approaches into parent-focused programs to increase their relevance and effectiveness across diverse communities at a broader level. Strengthening the capacity of families through coordinated community and policy-level efforts can develop a more resilient prevention system for OCSEA to protect our next generation.

### Limitations

While this systematic review provides valuable insights into the challenges parents face in preventing OCSEA, several limitations must be acknowledged. First, the review was limited to studies published between 2018 and 2025, potentially excluding relevant earlier works that could provide additional context or a broader historical perspective on parental challenges related to OCSEA. Furthermore, the geographical scope of the included studies varied, with a disproportionate representation from specific countries, which may limit the generalizability of the findings to other cultural and regional contexts. Second, variability in methodological approaches, sample sizes, and definitions of OCSEA across studies may have affected the consistency and reliability of the findings. Moreover, some studies primarily focused on the perspectives of mothers, which may introduce a gender bias and overlook the experiences and challenges faced by fathers and other caregivers. Third, the complexity of OCSEA, coupled with rapidly evolving technologies and platforms, presents an ongoing challenge that may not be fully captured in this review. The findings may become outdated as new trends and risks emerge in the digital landscape, necessitating continuous research in this area. Additionally, the review highlights subjective experiences of parents, often based on self-reported data. This reliance on personal narratives may introduce bias or underreporting of certain issues, particularly if parents feel uncomfortable discussing sensitive topics such as sexual abuse or exploitation.

Future research should aim to deepen the understanding of these interconnections to inform more effective strategies for supporting parents in their efforts to prevent OCSEA. By acknowledging these limitations, this review aims to provide a more nuanced understanding of the challenges in OCSEA prevention and emphasize the need for ongoing dialogue and research in this critical area.

## Conclusion

This review synthesized existing evidence on the challenges parents face in preventing OCSEA, revealing a complex interplay of barriers. We acknowledged that parents play an important role in OCSEA prevention and identified family-level challenges, including inadequate awareness, lack of knowledge, parental mediation struggles, and communication barriers, along with broader-level cultural norms and systemic gaps in OCSEA prevention. By understanding these challenges, we advocate for a more holistic and multi-level prevention system that supports parents as the first line of defense against OCSEA.

## Data Availability

The original contributions presented in the study are included in the article/Supplementary material, further inquiries can be directed to the corresponding author/s.
