# Supplementary material for: Challenges faced by parents in preventing online child sexual exploitation and abuse: a mixed methods systematic review
Source: Front Public Health. 2026 Feb 17;14:1765426. doi: 10.3389/fpubh.2026.1765426 (PMC12953457; doi:10.3389/fpubh.2026.1765426)
Supplement: Supplementary file 1 [file Table_1.docx]

**Supplementary File A**

**Search Strategies in All Databases**

| Ovid MEDLINE(R) |  |
| --- | --- |
| 1 | Child Abuse, Sexual/ |
| 2 | Pedophilia/ |
| 3 | ((child* or adolescen* or teen* or youth* or young* or minor? or underage* or toddler* or infant* or baby or babies or school*) adj3 (exploit* or extort* or "sexual* abus*" or "sexual* manipulat*" or "sexual offen*" or "sexual* assault*" or molest*)).mp. |
| 4 | P?edophil*.mp. |
| 5 | 1 or 2 or 3 or 4 |
| 6 | erotica/ |
| 7 | (porn or pornograph* or erotic*).mp. |
| 8 | ((sex* adj3 (text* or chat* or talk* or correspond* or contact* or interest*)) or sext*).mp. |
| 9 | Rape/ |
| 10 | (rape? or raping or rapist*).mp. |
| 11 | Sex Work/ |
| 12 | (prostitut* or "sex work*").mp. |
| 13 | ((sexual* or explicit) adj3 (photo* or image* or video* or material* or content*)).mp. |
| 14 | (nude? or nudity).mp. |
| 15 | sex* act*.mp. |
| 16 | ("sexual violence" or "sexual victim*").mp. |
| 17 | (cybersex* or "cyber sex*").mp. |
| 18 | or/6-17 |
| 19 | adolescent/ or exp child/ or infant/ or child, abandoned/ or child, adopted/ or child, exceptional/ or "child of impaired parents"/ or child, foster/ or child, orphaned/ or child, unwanted/ or minors/ or students/ |
| 20 | (child* or adolescen* or teen* or youth* or young* or minor? or underage* or toddler* or infant* or baby or babies or school*).mp. |
| 21 | 19 or 20 |
| 22 | 18 and 21 |
| 23 | 5 or 22 |
| 24 | internet/ or social media/ |
| 25 | (online* or internet* or web* or "social media" or facebook or twitter or instagram or "tik tok" or tiktok or tumblr or reddit or twitch or youtube or whatsapp or wechat or snapchat* or pinterest or quora or discord or mastodon or technolog* or digital* or virtual*).mp. |
| 26 | computers/ or computers, handheld/ or smartphone/ |
| 27 | (computer* or laptop* or ipad* or "smart phone*" or smartphone* or iphone* or android* or samsung).mp. |
| 28 | exp Cell Phone/ |
| 29 | ("cell phone*" or "text messag*" or email* or e-mail*).mp. |
| 30 | or/24-29 |
| 31 | exp parents/ or exp fathers/ |
| 32 | (parent* or father* or mother* or mom? or mum? or dad? or caregiver* or "care giver*" or guardian*).mp. |
| 33 | 31 or 32 |
| 34 | 23 and 30 and 33 |
| 35 | limit 34 to (english language and yr="2018 -Current") |

| Embase |  |
| --- | --- |
| 1 | child sexual abuse/ |
| 2 | pedophilia/ |
| 3 | pedophilia disorder/ |
| 4 | ((child* or adolescen* or teen* or youth* or young* or minor? or underage* or toddler* or infant* or baby or babies or school*) adj3 (exploit* or extort* or "sexual* abus*" or "sexual* manipulat*" or "sexual offen*" or "sexual* assault*" or molest*)).mp. |
| 5 | P?edophil*.mp. |
| 6 | 1 or 2 or 3 or 4 or 5 |
| 7 | exp erotica/ |
| 8 | (porn or pornograph* or erotic*).mp. |
| 9 | sexting/ |
| 10 | ((sex* adj3 (text* or chat* or talk* or correspond* or contact* or interest*)) or sext*).mp. |
| 11 | rape/ |
| 12 | (rape? or raping or rapist*).mp. |
| 13 | prostitution/ |
| 14 | (prostitut* or "sex work*").mp. |
| 15 | ((sexual* or explicit) adj3 (photo* or image* or video* or material* or content*)).mp. |
| 16 | (nude? or nudity).mp. |
| 17 | sex* act*.mp. |
| 18 | ("sexual violence" or "sexual victim*").mp. |
| 19 | (cybersex* or "cyber sex*").mp. |
| 20 | or/7-19 |
| 21 | juvenile/ or exp adolescent/ or exp child/ |
| 22 | minor (person)/ |
| 23 | (child* or adolescen* or teen* or youth* or young* or minor? or underage* or toddler* or infant* or baby or babies or school*).mp. |
| 24 | 21 or 22 or 23 |
| 25 | 20 and 24 |
| 26 | 6 or 25 |
| 27 | internet/ |
| 28 | social media/ |
| 29 | (online* or internet* or web* or "social media" or facebook or twitter or instagram or "tik tok" or tiktok or tumblr or reddit or twitch or youtube or whatsapp or wechat or snapchat* or pinterest or quora or discord or mastodon or technolog* or digital* or virtual*).mp. |
| 30 | computer/ or exp personal computer/ |
| 31 | exp mobile phone/ |
| 32 | (computer* or laptop* or ipad* or "smart phone*" or smartphone* or iphone* or android* or samsung).mp. |
| 33 | ("cell phone*" or "text messag*" or email* or e-mail*).mp. |
| 34 | or/27-33 |
| 35 | exp parent/ or exp adolescent parent/ or exp father/ or exp mother/ |
| 36 | (parent* or father* or mother* or mom? or mum? or dad? or caregiver* or "care giver*" or guardian*).mp. |
| 37 | 35 or 36 |
| 38 | 26 and 34 and 37 |
| 39 | limit 38 to (english language and yr="2018 -Current") |
| 40 | (conference or book or editorial or note or letter).pt. |
| 41 | 39 not 40 |

| APA PsycInfo |  |
| --- | --- |
| 1 | pedophilia/ |
| 2 | ((child* or adolescen* or teen* or youth* or young* or minor? or underage* or toddler* or infant* or baby or babies or school*) adj3 (exploit* or extort* or "sexual* abus*" or "sexual* manipulat*" or "sexual offen*" or "sexual* assault*" or molest*)).mp. |
| 3 | P?edophil*.mp. |
| 4 | 1 or 2 or 3 |
| 5 | pornography/ |
| 6 | (porn or pornograph* or erotic*).mp. |
| 7 | ((sex* adj3 (text* or chat* or talk* or correspond* or contact* or interest*)) or sext*).mp. |
| 8 | sexting/ |
| 9 | rape/ |
| 10 | (rape? or raping or rapist*).mp. |
| 11 | sex work/ |
| 12 | (prostitut* or "sex work*").mp. |
| 13 | ((sexual* or explicit) adj3 (photo* or image* or video* or material* or content*)).mp. |
| 14 | nudity/ or obscenity/ |
| 15 | (nude? or nudity).mp. |
| 16 | sex* act*.mp. |
| 17 | sexual violence/ |
| 18 | ("sexual violence" or "sexual victim*").mp. |
| 19 | cybersex/ |
| 20 | (cybersex* or "cyber sex*").mp. |
| 21 | or/5-20 |
| 22 | (child* or adolescen* or teen* or youth* or young* or minor? or underage* or toddler* or infant* or baby or babies or school*).mp. |
| 23 | 21 and 22 |
| 24 | 4 or 23 |
| 25 | internet/ |
| 26 | exp social media/ |
| 27 | (online* or internet* or web* or "social media" or facebook or twitter or instagram or "tik tok" or tiktok or tumblr or reddit or twitch or youtube or whatsapp or wechat or snapchat* or pinterest or quora or discord or mastodon or technolog* or digital* or virtual*).mp. |
| 28 | computers/ |
| 29 | exp mobile devices/ or exp mobile phones/ |
| 30 | (computer* or laptop* or ipad* or "smart phone*" or smartphone* or iphone* or android* or samsung).mp. |
| 31 | ("cell phone*" or "text messag*" or email* or e-mail*).mp. |
| 32 | or/25-31 |
| 33 | exp parents/ or exp expectant parents/ or exp fathers/ or exp mothers/ or exp single parents/ or exp parenting/ |
| 34 | (parent* or father* or mother* or mom? or mum? or dad? or caregiver* or "care giver*" or guardian*).mp. |
| 35 | 33 or 34 |
| 36 | 24 and 32 and 35 |
| 37 | limit 36 to (english language and yr="2018 -Current") |

| Database: CINAHL Plus with Full Text via EBSCOhost (1936 - Present) |  |
| --- | --- |
| S1 | (MH "Child Abuse, Sexual") |
| S2 | ((child* or adolescen* or teen* or youth* or young* or minor# or underage* or toddler* or infant* or baby or babies or school*) N3 (exploit* or extort* or "sexual* abus*" or "sexual* manipulat*" or "sexual offen*" or "sexual* assault*" or molest*)) |
| S3 | P#edophil* |
| S4 | S1 OR S2 OR S3 |
| S5 | (MH "Pornography") |
| S6 | (porn or pornograph* or erotic*) |
| S7 | (MH "Sexting") |
| S8 | ((sex* N3 (text* or chat* or talk* or correspond* or contact* or interest*)) or sext*) |
| S9 | (MH "Rape") OR (MH "Sexual Abuse") |
| S10 | (rape# or raping or rapist*) |
| S11 | (MH "Sex Work+") |
| S12 | (prostitut* or "sex work*") |
| S13 | ((sexual* or explicit) N3 (photo* or image* or video* or material* or content*)) |
| S14 | (nude? or nudity) |
| S15 | "sex* act*" |
| S16 | ("sexual violence" or "sexual victim*") |
| S17 | (cybersex* or "cyber sex*") |
| S18 | S5 OR S6 OR S7 OR S8 OR S9 OR S10 OR S11 OR S12 OR S13 OR S14 OR S15 OR S16 OR S17 |
| S19 | (MH "Adolescence+") OR (MH "Child+") OR (MH "Infant+") OR (MH "Minors (Legal)") OR (MH "Infant, Newborn+") |
| S20 | (child* or adolescen* or teen* or youth* or young* or minor# or underage* or toddler* or infant* or baby or babies or school*) |
| S21 | S19 OR S20 |
| S22 | S18 AND S21 |
| S23 | S4 OR S22 |
| S24 | (MH "Internet") OR (MH "Social Media+") OR (MH "World Wide Web") |
| S25 | (online* or internet* or web* or "social media" or facebook or twitter or instagram or "tik tok" or tiktok or tumblr or reddit or twitch or youtube or whatsapp or wechat or snapchat* or pinterest or quora or discord or mastodon or technolog* or digital* or virtual*) |
| S26 | (MH "Computers, Portable+") OR (MH "Computers, Hand-Held+") |
| S27 | (computer* or laptop* or ipad* or "smart phone*" or smartphone* or iphone* or android* or samsung) |
| S28 | (MH "Cellular Phone+") OR (MH "Text Messaging+") |
| S29 | ("cell phone*" or "text messag*" or email* or e-mail*) |
| S30 | S24 OR S25 OR S26 OR S27 OR S28 OR S29 |
| S31 | (MH "Parents+") OR (MH "Adolescent Parents+") OR (MH "Expectant Parents+") OR (MH "Fathers+") OR (MH "Mothers+") |
| S32 | (parent* or father* or mother* or mom# or mum# or dad# or caregiver* or "care giver*" or guardian*) |
| S33 | S31 OR S32 |
| S34 | S23 AND S30 AND S33 |
| S35 | Limit 34 to Publication Date 2018 - 2023, English language, Scholarly Peer-Reviewed Journals |

| Database: Scopus (1976 - Present) |
| --- |
| ( TITLE-ABS-KEY ( ( parent* OR father* OR mother* OR mom? OR mum? OR dad? OR caregiver* OR "care giver*" OR guardian* ) ) ) AND ( ( TITLE-ABS-KEY ( ( ( child* OR adolescen* OR teen* OR youth* OR young* OR minor? OR underage* OR toddler* OR infant* OR baby OR babies OR school* ) W/3 ( exploit* OR extort* OR "sexual* abus*" OR "sexual* manipulat*" OR "sexual offen*" OR "sexual* assault*" OR molest* ) ) ) OR TITLE-ABS-KEY ( p?edophil* ) ) OR ( ( TITLE-ABS-KEY ( ( porn OR pornograph* OR erotic* ) ) OR TITLE-ABS-KEY ( ( ( sex* W/3 ( text* OR chat* OR talk* OR correspond* OR contact* OR interest* ) ) OR sext* ) ) OR TITLE-ABS-KEY ( ( rape? OR raping OR rapist* ) ) OR TITLE-ABS-KEY ( ( prostitut* OR "sex work*" ) ) OR TITLE-ABS-KEY ( ( ( sexual* OR explicit ) W/3 ( photo* OR image* OR video* OR material* OR content* ) ) ) OR TITLE-ABS-KEY ( ( nude? OR nudity ) ) OR TITLE-ABS-KEY ( "sex* act*" ) OR TITLE-ABS-KEY ( ( "sexual violence" OR "sexual victim*" ) ) OR TITLE-ABS-KEY ( ( cybersex* OR "cyber sex*" ) ) ) AND TITLE-ABS-KEY ( ( child* OR adolescen* OR teen* OR youth* OR young* OR minor? OR underage* OR toddler* OR infant* OR baby OR babies OR school* ) ) ) ) AND ( TITLE-ABS-KEY ( ( online* OR internet* OR web* OR "social media" OR facebook OR twitter OR instagram OR "tik tok" OR tiktok OR tumblr OR reddit OR twitch OR youtube OR whatsapp OR wechat OR snapchat* OR pinterest OR quora OR discord OR mastodon OR technolog* OR digital* OR virtual* ) ) OR TITLE-ABS-KEY ( ( computer* OR laptop* OR ipad* OR "smart phone*" OR smartphone* OR iphone* OR android* OR samsung ) ) OR TITLE-ABS-KEY ( ( "cell phone*" OR "text messag*" OR email* OR e-mail* ) ) ) AND ( LIMIT-TO ( DOCTYPE , "ar" ) OR LIMIT-TO ( DOCTYPE , "re" ) ) AND ( LIMIT-TO ( LANGUAGE , "English" ) ) |

| Database: Cochrane library via Wiley (1992 - Present) |  |
| --- | --- |
| #1 | [mh ^"child abuse, sexual"] |
| #2 | [mh ^pedophilia] |
| #3 | ((child* or adolescen* or teen* or youth* or young* or minor? or underage* or toddler* or infant* or baby or babies or school*) NEAR/3 (exploit* or extort* or (sexual* NEXT abus*) or (sexual* NEXT manipulat*) or (sexual NEXT offen*) or (sexual* NEXT assault*) or molest*)):ti,ab,kw |
| #4 | P?edophil*:ti,ab,kw |
| #5 | {or #1-#4} |
| #6 | [mh ^erotica] |
| #7 | (porn or pornograph* or erotic*):ti,ab,kw |
| #8 | ((sex* NEAR/3 (text* or chat* or talk* or correspond* or contact* or interest*)) or sext*):ti,ab,kw |
| #9 | [mh ^rape] |
| #10 | (rape? or raping or rapist*):ti,ab,kw |
| #11 | [mh ^"sex work"] |
| #12 | (prostitut* or (sex NEXT work*)):ti,ab,kw |
| #13 | ((sexual* or explicit) NEAR/3 (photo* or image* or video* or material* or content*)):ti,ab,kw |
| #14 | (nude? or nudity):ti,ab,kw |
| #15 | (sex* NEXT act*):ti,ab,kw |
| #16 | (sexual NEXT (violence or victim*)):ti,ab,kw |
| #17 | (cybersex* or (cyber NEXT sex*)):ti,ab,kw |
| #18 | {or #6-#17} |
| #19 | [mh adolescent] or [mh child] or [mh minors] or [mh students] |
| #20 | (child* or adolescen* or teen* or youth* or young* or minor? or underage* or toddler* or infant* or baby or babies or school*):ti,ab,kw |
| #21 | #19 or #20 |
| #22 | #18 and #21 |
| #23 | #5 or #22 |
| #24 | [mh ^internet] or [mh ^"social media"] |
| #25 | (online* or internet* or web* or "social media" or facebook or twitter or instagram or "tik tok" or tiktok or tumblr or reddit or twitch or youtube or whatsapp or wechat or snapchat* or pinterest or quora or discord or mastodon or technolog* or digital* or virtual*):ti,ab,kw |
| #26 | [mh ^computers] or [mh ^"computers, handheld"] or [mh ^smartphone] |
| #27 | (computer* or laptop* or ipad* or (smart NEXT phone*) or smartphone* or iphone* or android* or samsung):ti,ab,kw |
| #28 | [mh "cell phone"] |
| #29 | ((cell NEXT phone*) or (text NEXT messag*) or email* or e-mail*):ti,ab,kw |
| #30 | {or #24-#29} |
| #31 | [mh parents] or [mh fathers] |
| #32 | (parent* or father* or mother* or mom? or mum? or dad? or caregiver* or (care NEXT giver*) or guardian*):ti,ab,kw |
| #33 | #31 or #32 |
| #34 | #23 and #30 and #33 |
