# Supplementary material for: Challenges faced by parents in preventing online child sexual exploitation and abuse: a mixed methods systematic review
Source: Front Public Health. 2026 Feb 17;14:1765426. doi: 10.3389/fpubh.2026.1765426 (PMC12953457; doi:10.3389/fpubh.2026.1765426)
Supplement: Supplementary file 2 [file Table_2.docx]

**Supplementary File B**

**Extraction Table for the Included Studies**

| **Authors and Year** | **Country** | **OCSEA type** | **Aim of Study** | **Study Design** | **Participants (parents only)** | **Findings** |
| --- | --- | --- | --- | --- | --- | --- |
| Adigwe (2024) | Nigeria | Sexting and Pornography | To investigate the gender perspectives of adolescents’ online risk-taking and parental mediation in Nigerian families. | Quantitative cross-sectional survey | 1,050 parents  (55% mothers and 45% fathers) | 1.Mothers tended to use restrictive and active mediation more for girls, while fathers employed technical mediation more often, but restrictive mediation overall was negatively correlated with online activities regardless of gender.  2. Girls spend more time online and were more exposed to contact and conduct risk |
| Ali et al. (2024) | Pakistan | Pornography | To examine parental perceptions of children’s Internet use and accidental exposure to pornography in Pakistan. | Qualitative interpretative study | 26 parents  (92% mothers and 8% fathers) | 1.Accidental exposure to online sexual content is often difficult to identify, as children may hide their experiences due to shyness.  2. Parents limited digital literacy and awareness of online risks hinder their ability to monitor children’s internet use, highlighting the need for stronger media literacy to recognize risks like cyberbullying, inappropriate content, and online predators. |
| Ali & Pasha (2024) | Pakistan | Pornography | To examine the parents' awareness of accidental  exposure to online indecent content among children. | Qualitative interpretative study | 26 parents  (77% mothers and 33% fathers) | 1.Most parents remain silent or avoid discussing accidental exposure to indecent online content with their children.  2. Parents demonstrate only moderate awareness of children’s accidental exposure to indecent online content, most often responding by monitoring internet history. |
| Allison (2018) | USA | Sexting and Pornography | 1. Examine parents’ level of awareness of early adolescent engagement in online behaviours and sexual risks via diverse technologies.  2. Explore parental protective practices aimed at mitigating online risks via smartphones, social media, and mobile applications among early adolescents ages 11 to 14 years.  3. Engage parents of early adolescents in identifying strategies that nursing professionals can use to counsel parents in addressing the concerns identified in Aims 1 & 2. | Mixed method study | 102 parents  (80% mothers and 20% fathers) | 1.Parents often struggle to talk about online risks, especially sexual risks, because of discomfort, limited knowledge, and lower digital expertise compared to their children.  2.Many parents rely on informal and inconsistent resources for guidance, but they need reliable and professional support to build knowledge, skills, and strategies for engaging with their children’s online activities.  3.Parents believe that nurses can play an important role by providing standardized tools and practical models to support education, skill-building, and sensitive conversations about online risks. |
| Corcoran et al. (2022) | USA | Sexting | To examine associations between parental media mediation and early adolescent sexting in a sample of U.S. youth and their parents. | Quantitative cross-sectional survey | 306 parents (parent-child dyads)  (60.8% mothers and 39.2% fathers) | 1.Restrictive parenting and active mediation were both linked to a lower likelihood of adolescents sending or receiving sexts, while technology controls showed no significant effect. |
| Dawson et al. (2024) | Ireland | Pornography | Identify the barriers to parent–child conversations about pornography and explore parents perceived barriers to talking to their children about pornography. | Mixed method study | 14 parents  (71.4% females and 28.6 males) | 1.Parents often avoid initiating discussions about pornography because of difficulty defining it in age-appropriate ways, anticipated embarrassment, or beliefs that adolescents would dismiss their advice.  2.Mothers frequently feel disconnected from their children’s sexual lives, while fathers of adolescent girls tend to see such discussions as the mother’s responsibility, leading to further communication gaps. |
| Dolev-Cohen & Ricon (2022) | Israel | Sexting | To identify parental characteristics that lead to dysfunctional communication (lower quality of communication) about sexting,  To determine whether parents perceived severity of sexting and the degree to which they perceive their adolescent to be susceptible to sexting function as mediating factors | Quantitative correlational study | 427 parents  (78.7% mothers and 21.3% fathers) | 1.Authoritarian and permissive parenting styles are associated with dysfunctional communication about sexting, while authoritative parenting is the only style that supports positive and open dialogue.  2.Parents often avoid discussing sexting because of embarrassment, technoference, or perceiving it as highly severe, which results in dysfunctional or absent communication. |
| Dorasamy et al. (2021) | Malaysia | Cyber Grooming | To investigate parents’ awareness of cyber grooming, specifically about adolescents.  To identify factors that can reduce cyber grooming and evaluate possible solutions for parents to play a role in building a bright society. | Qualitative interpretative study | 19 parents  (79% Mothers and 21% fathers) | 1.Parents recognize the importance of sex education but often feel discomfort and lack sufficient knowledge to discuss it, preferring schools to take on this responsibility.  2.Limited family time due to work-life constraints and the widespread accessibility of smartphones reduce opportunities for open communication, while many parents remain unaware of online grooming or fail to actively monitor their children’s online behavior. |
| Engelmann et al. (2025) | UK | Online Child Sexual Exploitation and Abuse | To understand how police, partner agencies, parents and children address OCSEA. Based on co-produced priorities, develop the first quality standards framework and approach aimed at preventing OCSEA offline, within local communities. | Locality-based mixed-methods design | 8 parents  (The percentage breakdown between mothers and fathers was not provided) | 1.Communication between parents and children about online risks is hindered by generational gaps, limited parental internet familiarity, and children’s fears of losing device access or being blamed, which discourage disclosure and help-seeking.  2.Both parents and children lack clarity about where to access support for OCSEA. |
| Gesser-Edelsburg & Abed Elhadi Arabia (2018) | Israel | Online Pornography | To characterize the barriers and difficulties that prevent sexual discourse in Arab society and enable pornography viewing according to the perceptions of adolescents and mothers. | Qualitative study | 20 parents (mothers only) | 1.Mothers often ignore pornography use among boys while strictly prohibiting it for girls, reflecting gender-based double standards.  2.Religious, cultural, and social taboos, along with embarrassment, fear of spousal disapproval, and lack of open discourse, create strong barriers to discussing sexuality within Arab families. |
| Gordon (2018) | USA | Sexting and Pornography | To investigate factors associated with parent-child communication about sexuality. | Mixed methods exploratory study | 163 parents  (The percentage breakdown between mothers and fathers was not provided) | 1.Same-gender parent-child conversations about sex are more common than cross-gender ones, but many barriers exist such as lack of knowledge, discomfort, limited time, cultural or language differences, and low parenting self-efficacy.  2.Parents often struggle to discuss sensitive topics like healthy relationships, body image, gender identity, and sexting, believing their children are too young or unresponsive. |
| Healy-Cullen et al. (2024) | New Zealand | Pornography | Determine how porn literacy education is viewed by those who will be most affected by it as stakeholders or end-users, drawing on a thematic analysis of interview data generated with 16 to 18-year-old students, parents, and teachers about porn literacy education and what it means to be “porn literate”. | Qualitative study | 7 parents  (71.4% Mothers and 28.6 fathers) | 1.Parents and teachers often view porn literacy education as protection against the harmful effects of pornography, but this risk-focused, moralistic, and top-down approach can oversimplify young people’s media use and shut down open dialogue.  2.Many parents view pornography as “dangerous” or “damaging,” reinforcing a deficit view of youth as naïve and overly vulnerable, which limits opportunities for youth agency and trust-based communication. |
| Lamprianidou et al. (2025) | Belgium | Sexting | To gain a deeper understanding of parents’ perspectives and practices regarding teen sexting.  To explore parents’ perspectives and practices regarding adolescent sexting in depth. And to explore how gendered dynamics shape these perspectives and practices, particularly in light of the often-cited double standard regarding girls’ and boys’ sexuality. | Qualitative study | 13 parents  (76.9% mothers and 23.1% fathers) | 1.Parents recognized gendered consequences of sexting shaped by social stereotypes, but most reported adopting the same rules for sons and daughters, striving for a gender-neutral approach.  2.Generational differences in technology use created challenges, as parents often struggled to understand sexting, trusted their children as “digital experts”, and found it difficult to balance autonomy with protection.  3.While parents generally viewed sexting as inauthentic intimacy compared to offline relationships, they expressed willingness to equip adolescents with tools to navigate risks. |
| Liu et al. (2021) | Taiwan and China | Pornography | To compare child and parental reports on childhood exposure to media violence/pornography and the impact on the mental health of children in Taiwan and China | Quantitative cross-sectional study | 2230 parents (parent-child dyads)  (69.8% mothers and 30.2% fathers) | 1.Fathers were generally less aware than mothers of their children’s exposure to violence or pornography, and overall parental awareness of such exposure on mobile devices was substantially lacking.  2.About 80% of parents were unaware of their children’s reported pornography exposure, with poor parent-child relationships, device ownership, and longer mobile use linked to greater unawareness. |
| Pandey & Reddy (2020) | India | Online Child Sexual Abuse | To explore the awareness, beliefs, and perceptions about Child Sexual Abuse (CSA) among the mothers from the lower economic sections of Delhi NCR. The study also aimed to understand how the mothers conceptualized CSA, specifically what acts they perceived to be a threat to children. | Qualitative exploratory study | 30 parents (Mothers only) | 1.Mothers had limited technological skills and education, leaving them unable to monitor their children’s online activities or respond effectively to suspected cases of online child sexual abuse.  2.Many held misconceptions, such as believing CSA is mostly perpetrated by men, that victims are usually female, that disabled people cannot abuse, and that only penetrative acts count as abuse, while underestimating non-penetrative and online forms. |
| Rudolph et al. (2022) | Australia and UK | Online Child Sexual Abuse | To examine the extent to which parents: discussed CSA relative to other sensitive topics with their children; used parenting behaviours expected to be protective against CSA; and mediated their child’s use of media. Parents’ attitudes towards CSA education were also quantified | Quantitative cross-sectional study | 248 parents  (87% mothers and 13% fathers) | 1.While parents practiced protective behaviours such as supervision and monitoring, their media mediation remained low to moderate, highlighting the need for greater awareness and strategies to manage children’s online risks.  2.Although most parents supported CSA prevention education and believed they should provide it, only about half had actually discussed sexual abuse with their children, often less than other sensitive topics. |
| Saleha et al. (2023) | Indonesia | Internet Child Sexual Abuse | To explore the decision-making process regarding the role of mothers in protecting their children from sexual harassment in the digital age. | Qualitative study | 12 parents (Mothers only) | 1.Mothers recognized the risks of internet exposure for both boys and girls and valued early sexual education, but many struggled with limited knowledge, cultural taboos, and difficulties in setting age-appropriate boundaries.  2.The COVID-19 pandemic and rapid technological developments intensified children’s online presence, creating new parenting challenges that require balancing supervision with children’s need for technology. |
| Speno & Halliwell (2023) | USA | Sexting | Investigate how parents discuss issues of sexting with their children, with a focus on the parental mediation strategies they may employ and the challenges they may experience in these discussions | Qualitative study | 23 parents  (65.2% mother and 34.8% fathers) | 1.Many parents avoided detailed discussions about sexting, either because they trusted their children “knew better” or because they lacked time, knowledge, and comfort with the topic, often feeling one step behind in technology.  2.Parents used discursive, investigative, and gatekeeping strategies, with discursive practices (open dialogue and examples) being most common, though conversations often focused on general privacy concerns rather than sexting directly. |
| Vall-Castelló et al. (2025) | Spain and Italy | Pornography | Analyze the knowledge, attitudes, and awareness of parents and teachers regarding young people’s internet usage and pornography consumption and their preparedness to discuss sexuality and pornography with their children or pupils | Quantitative cross-sectional study | 74 parents  (83.8% mothers and 16.2% fathers) | 1.Parents perceived boys as beginning pornography use earlier than girls, with mothers often taking the lead role in sexual education despite limited training in online safety or sexuality education.  2.Communication about sexuality can help reduce permissive attitudes and gender stereotypes linked to pornography, but embarrassment, cultural taboos, and parents’ own past experiences often prevent these discussions.  3.Parents recognized the harmful effects of pornography, but cross-cultural differences (e.g., Spanish vs. Italian parents) highlight the need for greater training and resources to better equip families and educators in addressing sexuality and online risks. |
